# LncRNA LINC00342 contributes to the growth and metastasis of colorectal cancer via targeting miR-19a-3p/NPEPL1 axis

**DOI:** 10.1186/s12935-020-01705-x

**Published:** 2021-02-15

**Authors:** Peng Shen, Lili Qu, Jingjing Wang, Quchen Ding, Chuanwen Zhou, Rui Xie, Honggang Wang, Guozhong Ji

**Affiliations:** 1 The Second Affiliated Hospital of Nanjing Medical University, Nanjing, 210000 China; 2 The Affiliated Huaian No.1 People’s Hospital of Nanjing Medical University, Huai’an, 223300 China

**Keywords:** Colorectal cancer, Invasion, LINC00342, Proliferation, miR-19a-3p, NPEPL1

## Abstract

**Background:**

Long intergenic non-protein coding RNA 00342 (LINC00342) has been identified as a novel oncogene. However, the functional role of LINC00342 in colorectal cancer (CRC) remains unclear.

**Methods:**

The expression of LINC00342 is detected by real-time PCR (RT-PCR) analysis. Cell proliferation, migration and invasion and xenograft model are examined to analyze the biological functions of LINC00342 in vitro and in vivo using colony formation, would healing and transwell analyses. Dual-luciferase reporter and RNA immunoprecipitation (RIP) assays are used to identify the target interactions between LINC00342, miR-19a-3p and aminopeptidase like 1 (NPEPL1).

**Results:**

LINC00342 was highly expressed in CRC. Down-regulation of LINC00342 inhibited cell proliferation and metastasis of CRC cells. Moreover, knocking down LINC00342 inhibited the tumor growth in vivo. Mechanistic investigation revealed that LINC00342 might sponge miR-19a-3p to regulate NPEPL1 expression. Further investigation indicated that the ontogenesis facilitated by LINC00342 was inhibited due to the depletion of NPEPL1.

**Conclusion:**

LINC00342 promotes CRC progression by competitively binding miR-19a-3p with NPEPL1.

## Highlights

1. LINC00342 is highly expressed in CRC tissues and cells.
2. Knockdown of LINC00342 inhibits CRC cell proliferation, migration and invasion in vitro.
3. LINC00342 acts as a ceRNA for miR-19a-3p to regulate NPEPL1 expression.

## Introduction

As the leading cause of cancer-related death worldwide, colorectal cancer causes over a half million deaths every year [1, 2]. Despite considerable progress and therapeutic strategies have been achieved in past decades, the mortality and 5-year survival rate of patients with CRC remain unsatisfactory [3]. Therefore, it is of great importance for us to understand the molecular mechanisms underlying CRC progression and identification of therapeutic biomarkers for improving the CRC patient prognosis and treatment.

Long non-coding RNAs (lncRNAs) are defined as a novel class of RNA molecules of length more than 200 nucleotides with narrow protein coding functions [4, 5]. Recently, a growing body of research has revealed that lncRNAs have been implicated in the tumorigenicity of multiple cancers, including CRC [6–8]. Dysregulated lncRNAs have been demonstrated to work as oncogenes or tumor suppressors through affecting a wide spectrum of biological activities, such as cell growth, invasion, metastasis and autophagy [9, 10]. For example, Wu et al. suggests that lncRNA TLN2-4 suppresses metastasis and is associated with patient survival in gastric cancer [11]. LncRNA SNHG1 has been identified functioning as a ceRNA to antagonize the effect of miR-145a-5p on the down-regulation of NUAK1 in nasopharyngeal carcinoma cell [12]. Besides, lncRNA PVT1 regulates ovarian cancer cell proliferation by increasing SOX2 expression [13]. However, to the best of our knowledge, the biological functions and the roles of long intergenic non-protein coding RNA 342 (LINC00342) in CRC hadn’t been reported yet.

MiRNAs are a class of small noncoding RNAs with a length of about 20–24 nucleotides. An increasing number of reports unveil that miRNAs play a key part in modulating the expression of diverse genes participating in the onset and progression of numerous cancers via regulating mRNA expression [14–16]. Previous studies have suggested that miR-223-3p promotes cell proliferation and invasion by targeting Arid1a in gastric cancer [17]. It is extensively accepted that miR-96 enhances the cervical cancer cell proliferation by targeting FOXO1 [18]. Additionally, miR-29a inhibits cell proliferation and migration by targeting the CDC42/PAK1 signaling pathway in cervical cancer [19]. Long et al. reports that miR-4319 suppresses colorectal cancer progression by targeting ABTB1 [20].

In this study, we demonstrated that LINC00342 was upregulated in CRC tissues and cells. Further study indicated that LINC00342 exerted its carcinogenic effects on the competing endogenous RNA (ceRNA) manner via miR-19a-3p/aminopeptidase like 1 (NPEPL1) axis. Therefore, our study was designed to investigate the interaction among LINC00342, miR-19a-3p and NPEPL1 in the regulation of CRC progression.

## Materials and methods

### Tissues

Fifty paired tumor tissues and adjacent non-tumor tissues were obtained from CRC patients during their hospitalization in our hospital between July 2016 and January 2018. The expressions of LINC00342 and miR-19a-3p were detected by real-time PCR.

### Cell lines

HCT-8, SW480, HT-29 and DLD-1, four human CRC cell lines and human colonic epithelial NCM460 cells were purchased from American Type Culture Collection (ATCC, Manassas, VA, USA). Cells were incubated in DMEM media supplemented with 10% fetal bovine serum (Invitrogen, Carlsbad, CA, USA) with 5% CO_2_ at 37 °C.

### Fluorescence in situ hybridization (FISH)

The slides of SW480 and HT-29 cells were placed in a 24-well plate at a density of 5 × 10^3^ cells/well, and cultured for 24 h. After washing with PBS, cells were fixed in 4% paraformaldehyde for 20 min, permeabilized by 0.5% Trition X-100 in PBS and digested with protein K. Then the hybridization was performed overnight at 37℃. The slides were finally stained with DAPI and observed under confocal laser microscopy (Zeiss, New York, NY, USA).

### Cell transfection

SW480 and HT-29 cells were transfected with empty vector, pcDNA3.1-LINC0342, mimic NC or miR-19a-3p mimic, synthesized by GenePharma Co., Ltd. (Shanghai, China) using Lipofectamine 2000 (Invitrogen, Carlsbad, CA, USA) following the manufacturer’s instructions, or were infected with lentivirus containing shRNA targeting LINC00342 or NPEPL1 or green fluorescent protein (GFP). Transfection efficiency was evaluated by real-time PCR and green fluorescence microscopy.

### Luciferase reporter assay

SW480 and HT-29 cells were co-transfected with the wild/mutated types of LINC00342 or NPEPL1 promoter reporters and miR-19a-3p mimic/mimic NC or pcDNA3.1-LINC00342. Luciferase assays were performed after 48-h transfection.

### RNA immunoprecipitation (RIP)

RIP assay was performed using the Immunoprecipitation Kit (Millipore, Billerica, MA, USA) according to the manufacturer’s instructions. Cell extracts of SW480 and HT-29 cells were incubated with protein A/G sepharose beads at 4 °C. Immunoprecipitated RNAs were subjected to real-time PCR.

### Cell proliferation

Cell proliferation was assessed using CCK-8 method and colony formation assay. For CCK-8 assay, cells were seeded into 96-well plates at a density of 2 × 10^3^ cells/well, and then CCK-8 dyes (Beyotime, Shanghai, China) were added into each group of cells for co-culture 24, 48 and 72 h at 37 °C. Then, the optical density (OD) values were measured. For colony formation assay, the colonies were counted after being stained with 0.5% crystal violet.

### Migration and invasion assay

For wound-healing assay, cells were wounded by a sterile micropipette tip, and photographed under a microscope (Olympus, Tokyo, Japan) at 0 h and 48 h after wounding. For transwell migration assay, cells were prepared in the upper chamber, while the lower chamber contained culture medium with serum. After 12-h culture, cells at the bottom were visualized under a microscope (×200 magnification) in 5 randomly selected fields of view after stained with 4% crystal violet. In the transwell invasion assay, filters coated with matrigel matrix were used.

### Real-time PCR analysis

Total RNA was extracted from tissues or cells using Trizol reagent and reverse-transcribed to cDNA. The quantitative analysis of LINC00342, miR-19a-3p and NPEPL1 expression were respectively normalized to GAPDH, U6 and GAPDH, determined using SYBR Premix Ex Taq (TaKaRa, Dalian, China). The specific primers were as followed: LINC00342 forward, 5′-CGTTCCAATGTGTTGGGT-3′and reverse, 5′-TGGGAGGAGGTTGAGATG-3′; miR-19a-3p forward, 5′-GGCGGGGAAAGTGTGTCT-3′and reverse, 5′-GTGCAGTCGTGGCGTGTG-3′; NPEPL1 forward, 5′-TCAGCCACACCCCAGATGGA-3′ and reverse: 5′-AGCCAAGCAGAACACAGCGT-3′; GAPDH forward, 5′-ACGGATTTGGTCGTATTGGGCG-3′ and reverse, 5′-CTCCTGGAAGATGGTGATGG-3′ and U6 forward, 5′-CTCGCTTCGGCAGCACA-3′ and reverse, 5′-AACGCTTCACGAATTTGCGT-3’.

### Western blot

After protein quantification, indicated antibodies (1:1000 dilution; Cell Signaling Technology) and horseradish peroxidase-conjugated secondary antibodies were applied for incubation. The target proteins were visualized using ECL system (7Sea PharmTech, Shanghai, China).

### Tumor xenograft model

BALB/c nude mice were purchased from Beijing Vitalriver Experimental Animal Technology Co., Ltd. All animal procedures were approved by Animal Care Committee of the Second Affiliated Hospital of Nanjing Medical University. HT-29 cells (1 × 10^6^) stably transfected with sh-LINC00342 or sh-NC were subcutaneously injected into nude mice. The experimental mice were routinely monitored and sacrificed on day 25 by cervical dislocation and their tumors were collected. Tumor volume was measured every 5 days and was calculated by the following formula: volume (mm^3^) = (length × width^2^)/2.

### Hematoxylin and eosin (H&E) staining

The tumor tissues were fixed in 4% paraformaldehyde and then embedded in paraffin. The sections were stained with H&E. At least three different sections of tumor tissues were examined for each group using a microscope to assess the histopathological alterations.

### Immunohistochemistry (IHC) assay

Experimental method was as described above [8]. Briefly, lung samples were fixed and cut into 4 μm thick sections, dried, deparaffinized, and dehydrated in a graded ethanol series, and finally incubated with H_2_O_2_. Blocked with bovine serum, sections were incubated overnight with monoclonal antibodies (1: 1000 dilutions) of Ki67, E-cadherin and Vimentin. The secondary antibody (1: 500 dilutions) was used to incubate the sections, and then the sections were incubated with horseradish peroxidase-conjugated streptavidin. A 3, 3′-diaminobenzidine (DAB) substrate kit was used, and slides were counterstained with hematoxylin. The section was visualized under a light microscope (Olympus Corporation, Tokyo, Japan).

### Statistical analysis

Data were expressed as mean ± standard deviation (SD). Comparisons between groups were assessed by two-sided Student’s t test or analysis of variance using the SPSS 22.0 (IBM, Armonk, NY, USA). *P* < 0.05 was considered statistically significant.

## Results

### LINC00342 is significantly up-regulated in CRC

**Fig. 1:**
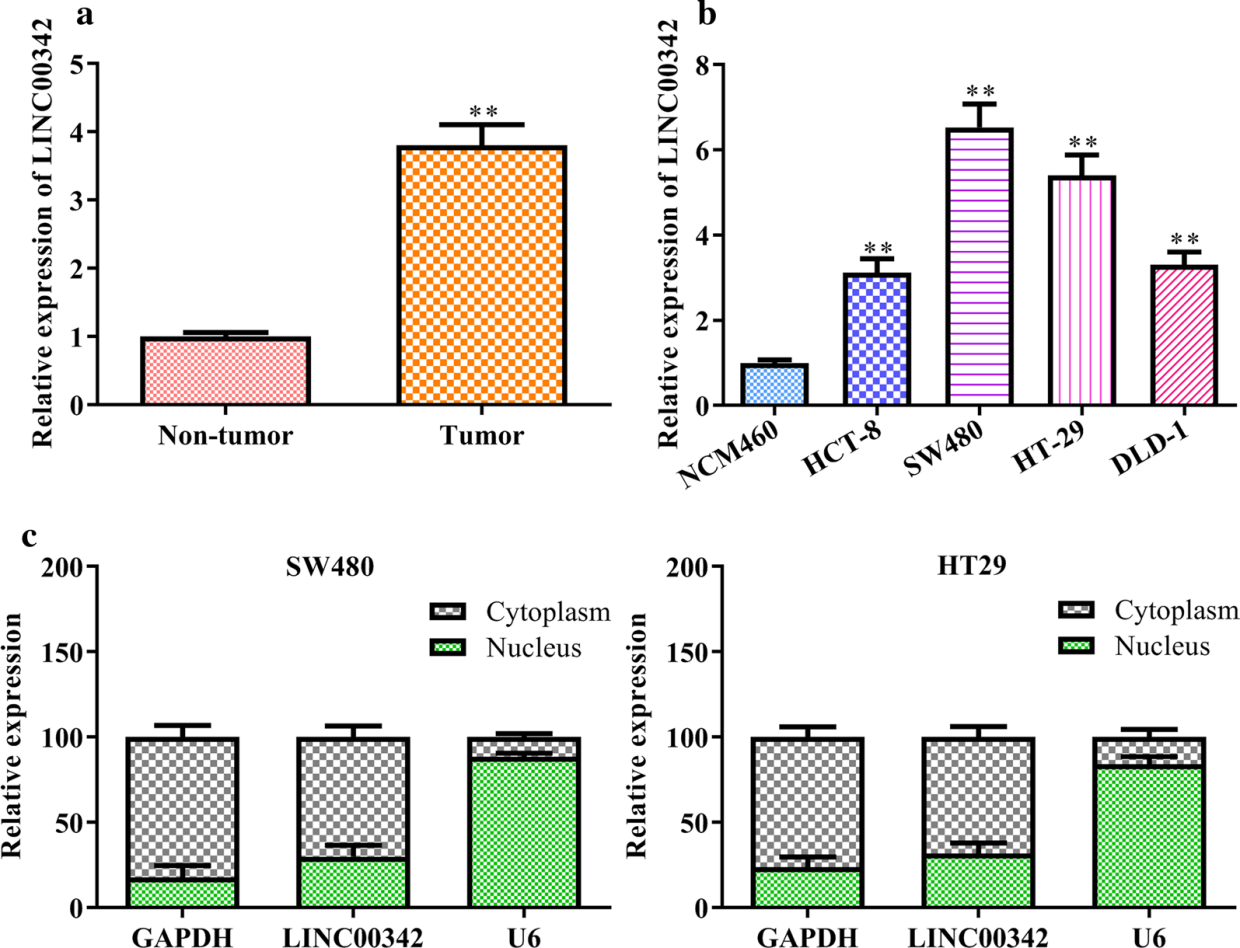
LINC00342 is significantly upregulated in CRC tissues and cells. **a** Expression of LINC00342 in non-tumor tissues and CRC tumor tissues determined by real-time PCR; **b** expression of LINC00342 in colonic epithelial NCM460 cells and four CRC cells (HCT-8, SW480, HT-29 and DLD-1); **c** subcellular fractionation assay of LINC00342 in SW480 and HT-29 cells, analyzed by RNA-FISH. **P < 0.05 vs. the non-tumor group or NCM460 cells

Using real-time PCR analysis, the increased expression of LINC00342 was observed in CRC tumor tissues and cell lines (*P* < 0.01, Fig. 1a, b). Analyzed by RNA-FISH, LINC00342 was distributed in the cytoplasm of CRC cells (Fig. 1c).

### LINC00342 requires for CRC cell proliferation, migration and invasion

**Fig. 2:**
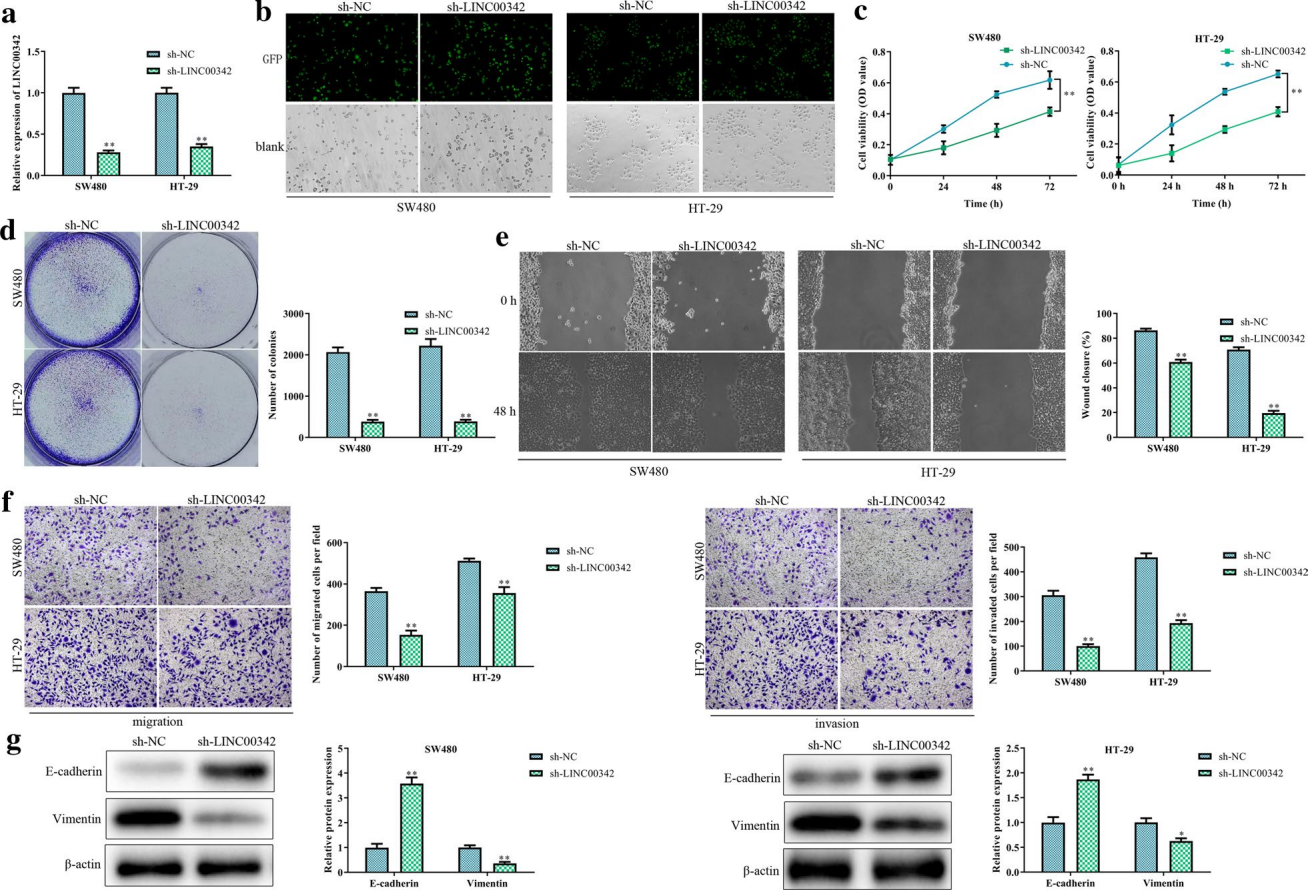
LINC00342 requires for the proliferation, migration and invasion of CRC cells. SW480 and HT-29 cells were infected with lentivirus containing sh-NC or sh-LINC00342. The transfection efficiency was confirmed by real-time PCR (**a**) and green fluorescence microscopy (**b**); cell proliferation was measured with CCK-8 (**c**) and colony formation (**d**) assays; cell migration and invasion was determined by wound healing assay (**e**) and transwell migration and invasion assays (**f**); the protein levels of E-cadherin and Vimentin were measured by western blotting (**g**). *P < 0.05, **P < 0.01 vs. sh-NC group

To investigate the biological functions of LINC00342 in CRC cells, specific shRNA was used to knock down LINC00342 expression in SW480 and HT-29 cells, and real-time PCR (Fig. 2a) and green fluorescence microscopy (Fig. 2b) were performed to confirm the transfection efficiency. The results showed that the cell viability (*P* < 0.01, Fig. 2c) and colony formation abilities (*P* < 0.01, Fig. 2d) were inhibited by LINC00342 silencing. The wound healing assay and the transwell assays indicated that suppression of LINC00342 expression significantly inhibited cell migration and invasion (*P* < 0.01, Fig. 2e, f). Moreover, the protein levels of E-cadherin were largely increased, while Vimentin expression was observably reduced after LINC00342 silencing (*P* < 0.01, Fig. 2g).

MiR-19a-3p is down-regulated in CRC and negatively regulated by LINC00342.

**Fig. 3:**
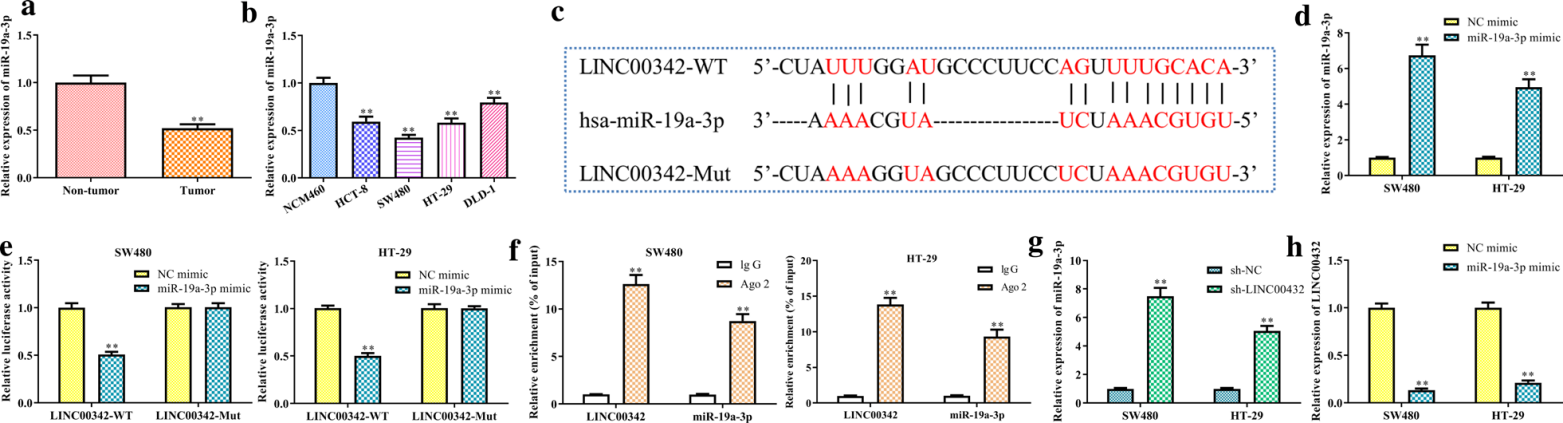
MiR-19a-3p is downregulated in CRC and negatively regulated by LINC00342. Expression of miR-19a-3p in CRC tumor tissues (**a**) and cell lines (**b**); ^**^P < 0.01 *vs*. the non-tumor group or NCM460 cells. The miRNA target sites of LINC00342 was predicted by using DIANA tools, the miR-19a-3p binding site and the mutational LINC00342 sequence were shown (**c**); expression of miR-19a-3p in SW480 and HT-29 cells transfected with miR-19a-3p mimic or mimic NC (**d**); SW480 and HT-29 cells were co-transfected with the wild or mutant type LINC00342 plasmids and miR-19a-3p mimic or mimic NC, and the luciferase activities were measured (**e**); **P < 0.01 vs. NC mimic group. RIP assays were performed in SW480 and HT-29 cells, and precipitated RNA levels were presented as fold changes in Ago2 relative to IgG immunoprecipitates (**f**); **P < 0.01 vs. IgG group. Expression of miR-19a-3p in SW480 and HT-29 cells transfected with sh-NC or sh-LINC00342 (**g**); **P < 0.01 vs. sh-NC group. Expression of LINC00342 in SW480 and HT-29 cells transfected with NC-mimic or miR-19a-3p mimic (**h**); **P < 0.01 vs. NC mimic group

As shown in Fig. 3a, b, miR-19a-3p was lowly expressed in CRC tissues and cells. Furthermore, miR-19a-3p was predicted as a target of LINC00342 by DIANA tools (http://carolina.imis.athena-innovation.gr/) (Fig. 3c). Then, dual-luciferase reporter assay demonstrated that miR-19a-3p overexpression after transfection with miR-19a-3p mimic (*P* < 0.01, Fig. 3d) significantly reduced the relative luciferase activity of LINC00342-WT in SW480 and HT-29 cells (*P* < 0.01, Fig. 3e). RIP assay indicated that LINC00342 and miR-19a-3p were evidently enriched in Ago2 immunoprecipitates in contrast to IgG immunoprecipitates (*P* < 0.01, Fig. 3f). In addition, knockdown of LINC00342 promoted the miR-19a-3p expression in CRC cells (*P* < 0.01, Fig. 3g).

### MiR-19a-3p inhibits CRC cell proliferation, migration and invasion

**Fig. 4:**
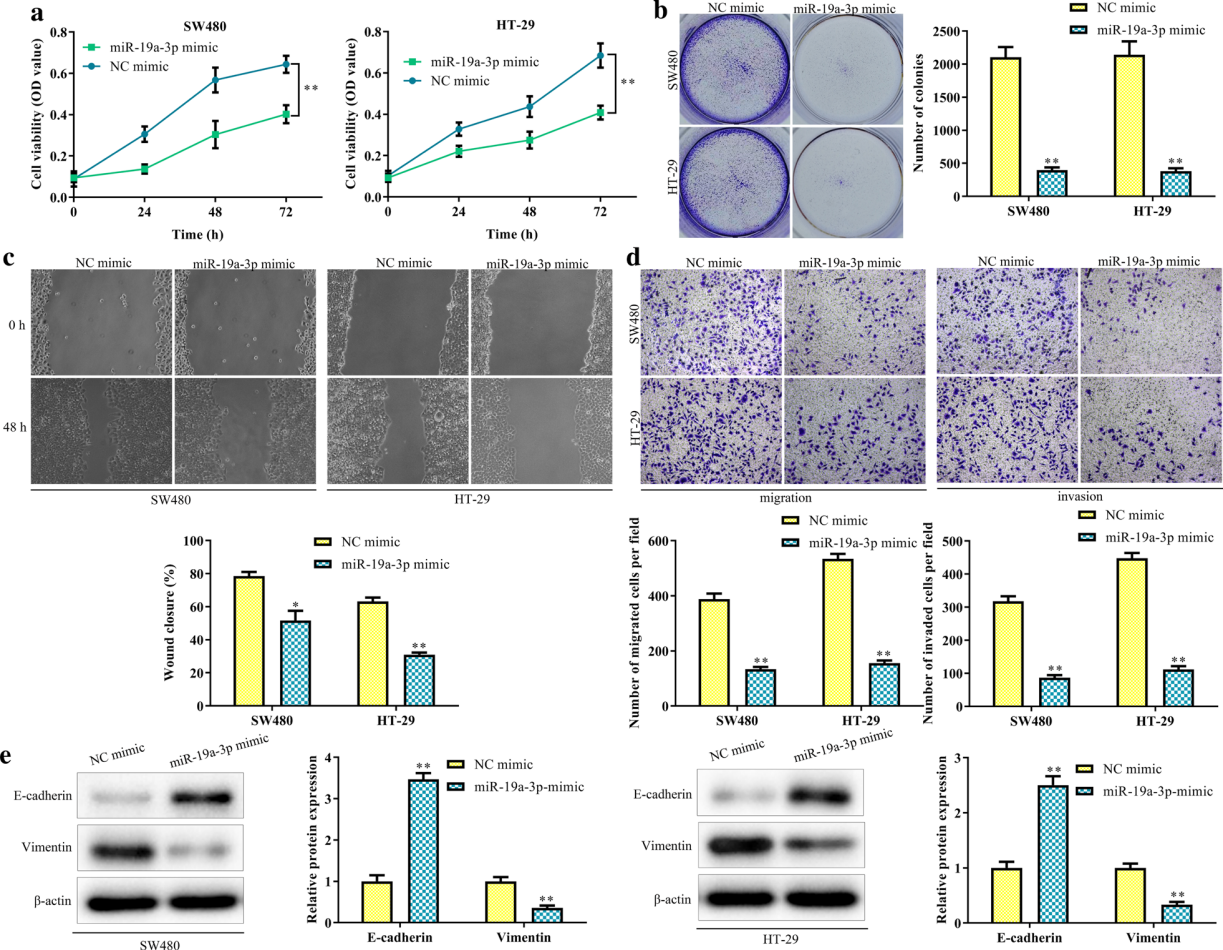
MiR-19a-3p inhibits CRC cell proliferation, migration and invasion. The proliferation (**a**, **b**), migration and invasion abilities (**c**, **d**) and the protein levels of E-cadherin and Vimentin (E) in SW480 and HT-29 cells transfected with mimic NC or miR-19a-3p mimic. **P < 0.01 vs. NC mimic group

We explored the biological function of miR-19a-3p in CRC. Ectopic expression of miR-19a-3p memorably reduced cell proliferation (*P* < 0.01, Fig. 4a) and colony formation (*P* < 0.01, Fig. 4b) in SW480 and HT-29 cells. The migration and invasion abilities of CRC cells were diminished after up-regulation of miR-19a-3p (*P* < 0.01, Fig. 4c, d). Thereafter, overexpression of miR-19a-3p significantly increased E-cadherin expression but suppressed the protein levels of Vimentin (*P* < 0.01, Fig. 4e).

### LINC00342 functions as a ceRNA of NPEPL1 by sponging miR-19a-3p

**Fig. 5:**
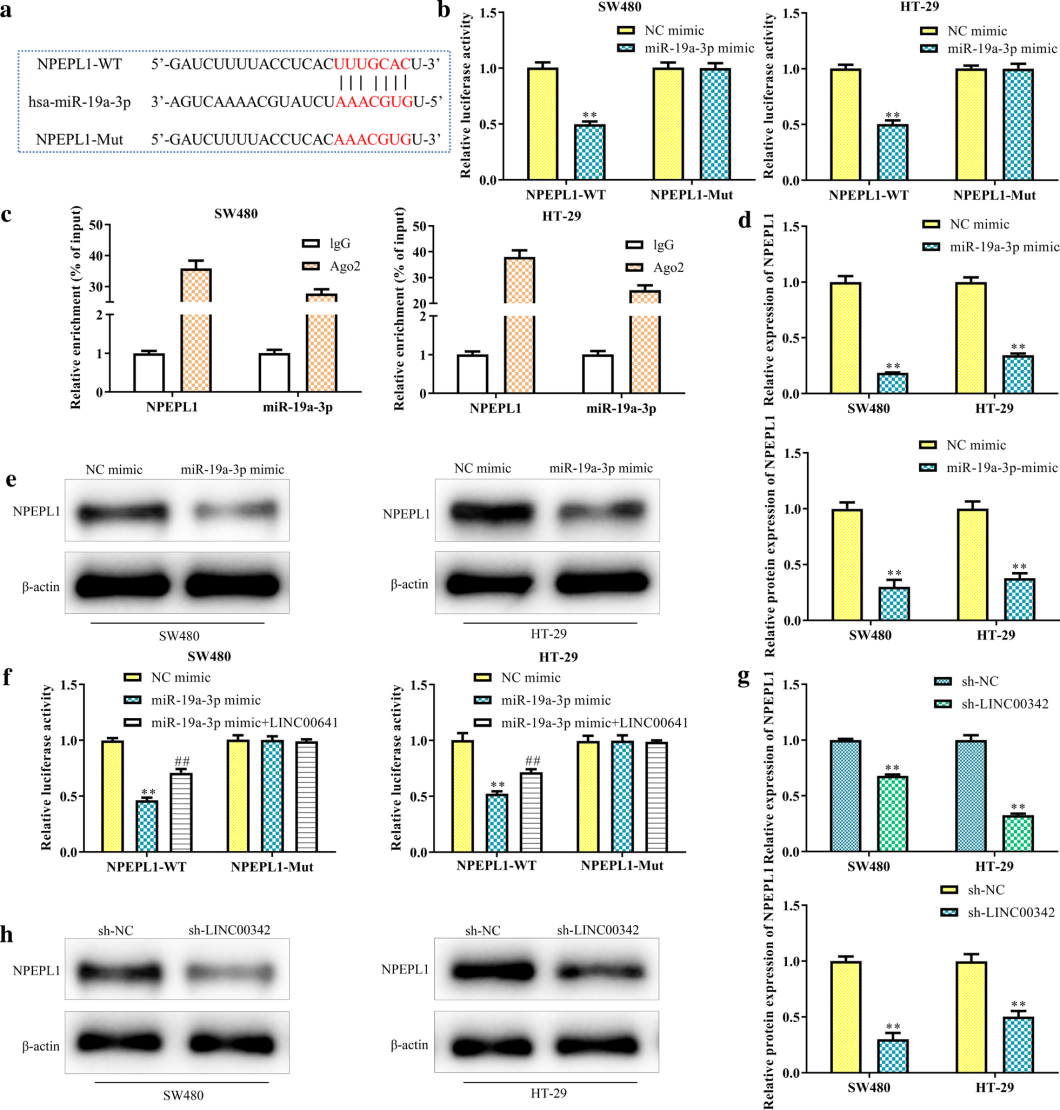
LINC00342 functions as a ceRNA of NPEPL1 by sponging miR-19a-3p. TargetScan was used to predict that NPEPL1 mRNA 3′UTR contains miR-19a-3p binding site, and the mutational 3′UTR of NPEPL1 mRNA were indicated (**a**); SW480 and HT-29 cells were co-transfected with the wild or mutant type NPEPL1 plasmids and miR-19a-3p mimic or mimic NC, and the luciferase activities were measured (**b**); ^**^P < 0.01 *vs*. mimic NC group. RIP assay showed that NPEPL1 was a direct target of miR-19a-3p (**c**); The mRNA and protein levels of NPEPL1 in SW480 and HT-29 cells transfected with mimic NC or miR-19a-3p mimic (**d**, **e**); **P < 0.01 vs. mimic NC group. Luciferase reporter assay showed the interaction between LINC00342, miR-19a-3p and NPEPL1 (**f**); **P < 0.01 vs. NC mimic group. ^##^*P* < 0.05 vs. miR-19a-3p mimic group. The mRNA and protein levels of NPEPL1 in SW480 and HT-29 cells transfected with sh-NC or sh-LINC00342 (**g**, **h**); **P < 0.01 vs. sh-NC group

Predicted by TargetScan (http://www.targetscan.org), NPEPL1 was selected as a candidate target due to the carcinogenic role of miR-19a-3p in several cancers (Fig. 5a). Luciferase assay in SW480 and HT-29 cells confirmed that overexpression of miR-19a-3p strikingly reduced the luciferase activity of NPEPL1-WT (*P* < 0.01, Fig. 5b), indicating that miR-19a-3p directly bound to NPEPL1 in CRC cells, which was verified by RIP experiments (*P* < 0.01, Fig. 5c). Moreover, we found that the mRNA and protein expression of NPEPL1 was reduced by miR-19a-3p mimic in SW480 and HT-29 cells (*P* < 0.01, Fig. 5d, e). Luciferase assays showed that the reduced luciferase activity of NPEPL1-WT, induced by overexpression of miR-19a-3p was eliminated by LINC00342 (*P* < 0.01, Fig. 5f). The downregulated NPEPL1 expression at mRNA and protein levels were observed after knockdown of endogenous LINC00342 (*P* < 0.01, Fig. 5g, h).

### Knockdown of NPEPL1 rescues the carcinogenesis of LINC00342 on CRC progression

**Fig. 6:**
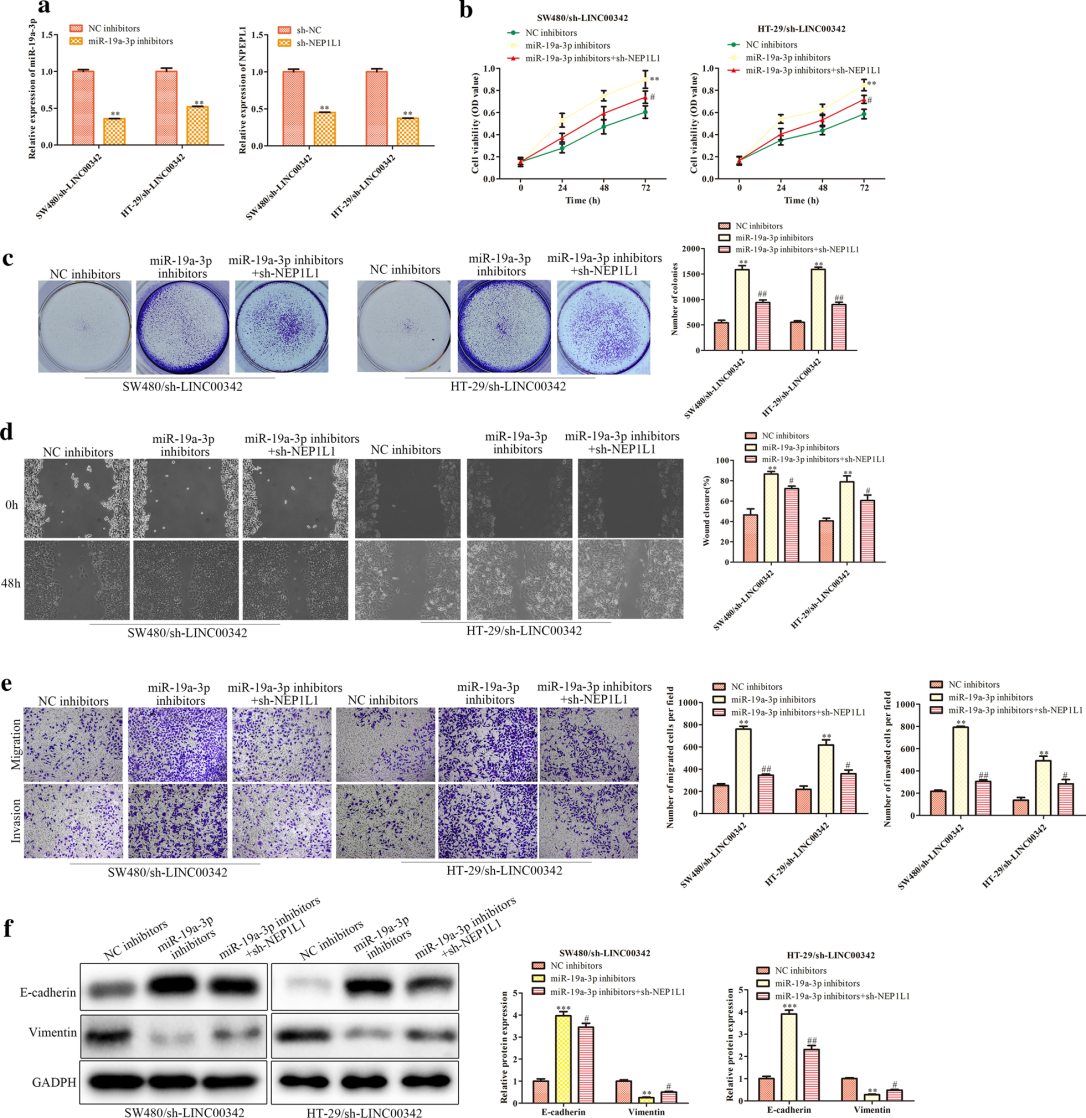
Knockdown of NPEPL1 rescues the carcinogenesis of LINC00342 on CRC progression. The expression of NPEPL1 and LINC00342 in NPEPL1 and LINC00342-overexpressed SW480 cells (**a**); **P < 0.01 vs. NC inhibitor group. The proliferation (**b**, **c**), migration and invasion abilities (**d**, **e**) and the protein levels of E-cadherin and Vimentin (**f**) in SW480 and HT-29 cells transfected with LINC00342 and/or sh-NPEPL1; **P < 0.01, ***P < 0.001 vs. NC inhibitor group; ^#^*P* < 0.05, ^##^*P* < 0.05 vs. miR-19a-3p inhibitor + sh-NC group

Rescue experiments were performed in SW480 cells by transfection with LINC00342 and/or sh-NPEPL1 to investigate whether LINC00342-induced carcinogenesis were mediated by miR-19a-3p/NPEPL1 axis in CRC cells, (Fig. 6a). The proliferation (*P* < 0.01, Fig. 6b, c) migration and invasion capacities (*P* < 0.01, Fig. 6d, e) were elevated by LINC00342 overexpression, but repressed by NPEPL1 down-regulation. Additionally, restoration of LINC00342 expression markedly inhibited E-cadherin expression, but increased Vimentin levels, whereas NPEPL1 silencing led to an opposite effect. However, the pro-tumor effects of LINC00342 were reversed by NPEPL1 knockdown (*P* < 0.01, Fig. 6f).

### *Silencing LINC00342 inhibits tumorigenesis of CRC cells through *in vivo

**Fig. 7:**
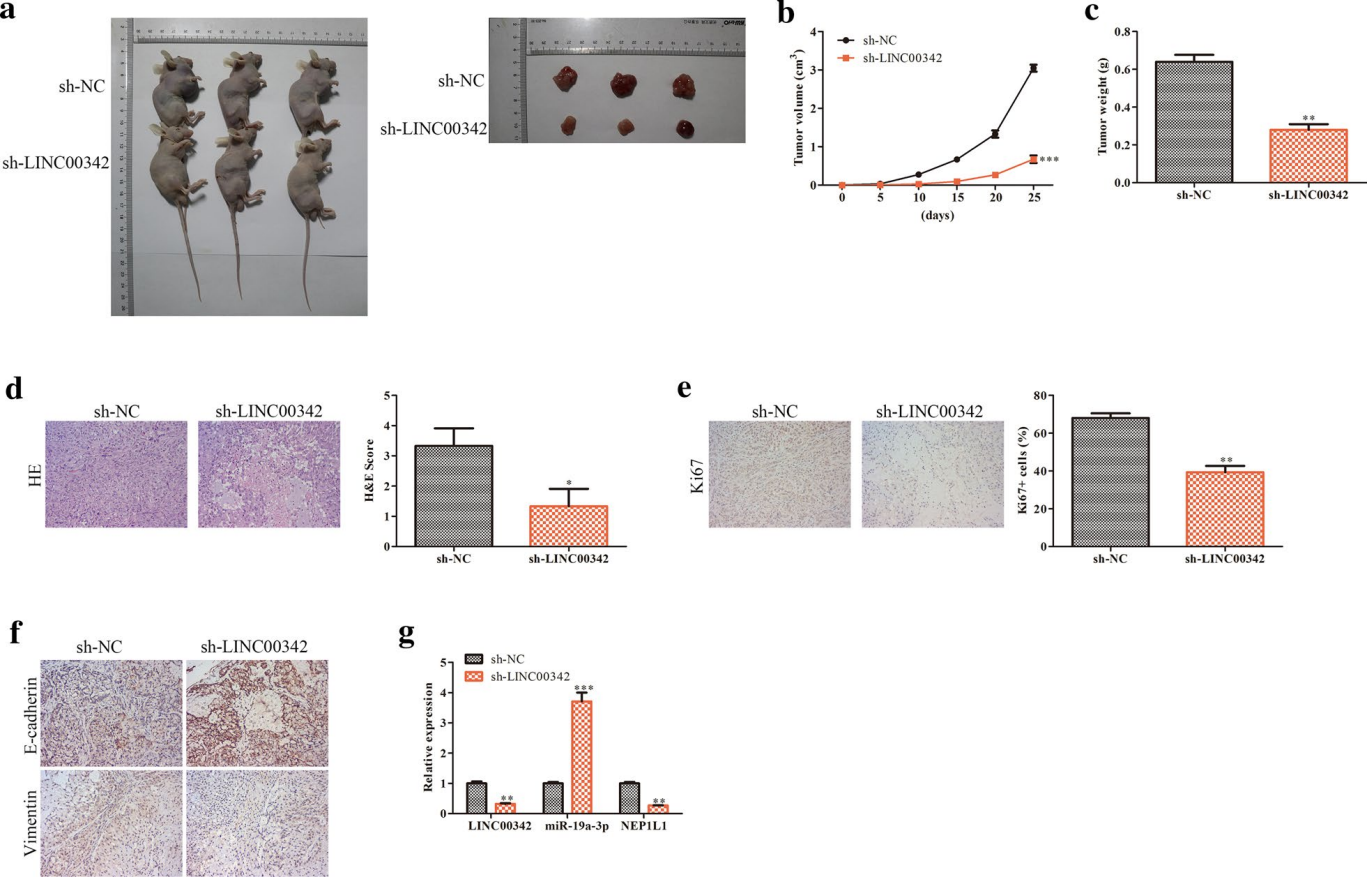
Silencing LINC00342 inhibits tumorigenesis of CRC cells through in vivo. Representative images of nude mice and tumors (**a**); the growth curves of tumors derived from HT-29 cells transfected with sh-LINC00342 or si-NC (**b**); the average weight of tumors (**c**); the analysis of histological in tumor sections (**d**); immunohistochemistry assay was conduct to assess the expression levels of Ki67, E-cadherin and vimentin. The photographs were taken at the magnification of × 200 (E, F). *P < 0.05, **P < 0.01, ***P < 0.001 vs. sh-NC group

Xenograft nude mouse models were established in order to elucidate the role of LINC00342 in CRC in vivo. The results showed that LINC00342 knockdown exhibited lower tumor growth compared with sh-NC group (*P* < 0.001, Fig. 7a, b). Moreover, compared with the sh-NC, tumor weight was reduced by LINC00342 silencing (*P* < 0.01, Fig. 7c). In addition, with the analysis of histological and IHC in tumor sections, the expression levels of Ki67 and vimentin were higher, while the E-cadherin expression level was lower in the sh-LINC00342 group than the sh-NC group (*P* < 0.01, Fig. 7d–f). Hence, these findings indicated that knockdown of LINC00342 inhibited the tumor growth in vivo.

## Discussion

The critical functions of lncRNAs have been reported in CRC development. For instance, MALAT1 expression is correlated with the maintenance of CRC stem cell properties [21]. LncRNA PCAT6 modulates 5-fluorouracil chemoresistance in CRC cells through a miR-204/HMGA2 pathway [22]. LncRNA HOTAIR expression is associated with the aggressive biological behaviors of CRC cells [23]. In our research, we found that LINC00342 was up-regulated in CRC. Functional analysis revealed that LINC00342 silencing inhibited proliferation, migration and invasion in vitro and in vivo in CRC. Moreover, LINC00342 functioned as a tumor promoter by sponging miR-19a-3p and releasing NPEPL1. Taken together, our study illuminated that a LINC00342/miR-19a-3p/NPEPL1 axis existed in CRC.

LINC00342, a novel lncRNA located on chromosome 2q11.1, is up-regulated in non-small cell lung cancer (NSCLC) and infantile hemangiomas [24, 25]. High expression of LINC00342 predicts a poor prognosis in NSCLC [26]. Knockdown of LINC00342 inhibits NSCLC cell proliferation, migration and invasion [27]. However, the expression and function of LINC00342 remained unknown in CRC. Herein, LINC00342 was highly expressed in CRC tissues and cells. Our study further demonstrated that LINC00342 induced the proliferation and metastasis of CRC cells and tumor growth. These findings suggested that LINC00342 acted as an oncogene in CRC.

Increasing evidence shows a novel regulatory mechanism that lncRNAs serve as miRNA sponges at a post-transcriptional level in the occurrence and development of various tumors. For example, Xu et al. has reported that lncRNA TUSC7 functions as a miR-211 decoy in the regulation of CDK6SH3 and suppresses the proliferation of CRC cells [28]. Li et al. has exhibited that lncRNA MFI2-AS1 regulates CRC cell proliferation, metastasis, cell cycle distribution, and apoptosis through competitively interacting with miR-574-5p to upregulate MYCBP expression [29]. Shi et al. finds that ZNFX1-AS1 serves as a ceRNA to upregulate EZH2 expression by sponging miR-144 to promote CRC progression [30]. In our study, we discovered that LINC00342 was involved in the ceRNA regulatory network and functioned as an endogenous miRNA sponge to bind to miR-19a-3p and regulated its function. In line with our finding, Chen et al. reports that ectopic LINC00342 expression facilitates NSCLC cell proliferation, migration, and invasion by sponging miR-203a-3p [27].

MiR-19a-3p functioned as an oncogene or a tumor suppressor in various cancers. For example, miR-19a-3p is distinctly increased in hepatocellular carcinoma (HCC), and promotes HCC cell metastasis and chemoresistance [31]. In contrast, miR-19a-3p expression is reduced in osteosarcoma, and silencing miR-19a-3p suppresses osteosarcoma cell proliferation and induces apoptosis [32]. In addition, miR-19a-3p is reported to be down-regulated and to further inhibit the angiogenesis of CRC cells [33]. However, how miR-19a-3p was controlled in CRC remained unclear.

In this study, enforced overexpression of miR-19a-3p reduced the capabilities of proliferation, migration and invasion of CRC cells, suggesting that miR-19a-3p might play a tumor-suppressive role in CRC. Bioinformatics prediction and luciferase reporter assays confirmed that a direct binding existed between LINC00342 and miR-19a-3p. In addition, the oncogene NPEPL1 was demonstrated to be a direct target of miR-19a-3p, which was consistent with the previous research [34]. Functional analyses revealed that the overexpression of LINC00342 partly overturned the tumor suppressive effects of NPEPL1 knockdown in CRC, indicating that LINC00342 accelerated CRC progression, at least in part, through regulating the expression of NPEPL1 by serving as a sponge of miR-19a-3p.

## Conclusion

In summary, our study for the first time revealed a novel LINC00342-miR-19a-3p-NPEPL1 regulatory network in the pathogenesis and development of CRC: LINC00342 promoted CRC progression by competitively binding miR-19a-3p with NPEPL1. The limitation of this study was that the exploration on the mechanism of LINC00342 was not deep enough. We would further investigate the mechanism and network of LINC00342 in the future work.

## Data Availability

The raw data supporting the conclusions of this manuscript will be made available by the authors, without undue reservation, to any qualified researcher.
